# Comparison of Conventional Methods and Laser-Assisted Rapid Prototyping for Manufacturing Fixed Dental Prostheses: An In Vitro Study

**DOI:** 10.1155/2015/318097

**Published:** 2015-10-21

**Authors:** Giorgio Pompa, Stefano Di Carlo, Francesca De Angelis, Maria Paola Cristalli, Susanna Annibali

**Affiliations:** ^1^Department of Oral and Maxillofacial Sciences, “Sapienza” University of Rome, 00161 Rome, Italy; ^2^Department of Medico-Surgical Sciences and Biotechnologies, “Sapienza” University of Rome, 00161 Rome, Italy

## Abstract

This study assessed whether there are differences in marginal fit between laser-fusion and conventional techniques to produce fixed dental prostheses (FDPs). A master steel die with 2 abutments was produced to receive a posterior 4-unit FDPs and single copings. These experimental models were divided into three groups (*n* = 20/group) manufactured: group 1, Ni-Cr alloy, with a lost-wax casting technique; group 2, Co-Cr alloy, with selective laser melting (SLM); and group 3, yttria-tetragonal zirconia polycrystal (Y-TZP), with a milling system. All specimens were cut along the longitudinal axis and their adaptation was measured at the marginal and shoulder areas on the right and left sides of each abutment. Measurements were made using a stereomicroscope (×60 magnification) and a scanning electron microscope (×800 magnification). The data were analyzed using one-way analysis of variance and the Bonferroni post hoc test, with a significance cutoff of 5%. Significant differences (*P* < 0.05) were observed between group 3 and the other groups. The marginal opening was smallest with Co-Cr alloy substructures, while the shoulder opening was smallest with Ni-Cr alloy substructures. Within the limitations of this study, the marginal fit of an FDP is better with rapid prototyping (RP) via SLM than conventional manufacturing systems.

## 1. Introduction

Fixed dental prostheses (FDPs) are the rehabilitation of choice after endodontic and operative treatments, especially among all over the world [1]. Moreover, the introduction of implant restorations has increased the popularity of fabricating crowns and bridges to rehabilitate the edentulous area. The development of both casting gold alloys and dental precision casting systems has contributed to the application of metallic restorations. However, patients are increasingly requesting metal-free restorations for aesthetics and biosafety reasons [2].

A poor marginal fit of crowns is responsible for 10% of prosthetic failures; these failures are mainly due to secondary caries, periodontal diseases, pulpitis, necrosis, and technical errors [3, 4]. Clinical trials have demonstrated the importance of the marginal fit in the quality assessment of fixed restorations and for their clinical success [5]. There are various opinions about the required marginal fit in the literature [6–9], but gaps of 100–150 *μ*m are generally considered to be clinically acceptable [8, 10–14]. The marginal fit of FDPs is a fundamental requirement [15, 16] to achieve a clinically acceptable result with a good long-term prognosis [17–20].

Noble-metal alloys are generally preferred to base-metal alloys for the manufacture of FDPs due to their biocompatibility, good mechanical properties, and excellent ceramic-metal bonding [21]. However, nonprecious alloys, such as nickel-chromium (Ni-Cr) and cobalt-chromium (Co-Cr), are now commonly used for the substructure of metal-ceramic restorations due to economic considerations [22, 23]. The trend in modern dentistry is to use metal-free restorations, but metal-ceramic crowns are still the most widely used ones due to their excellent mechanical properties and clinical performance, low cost compared to metal-free restorations, simple cementation technique, and role in natural reproduction of lost dentition in most restorative treatments [24]. The conventional technique for fabricating a metal structure is lost-wax casting. However, computer-aided design and manufacturing (CAD/CAM) technologies now allow the precise design of elements produced by specialized computerized equipment [25]. The use of CAD/CAM systems has been considered by several authors in the dentistry field, especially for the manufacture of FDPs [26–34]. Laser-assisted rapid prototyping (RP) is a CAD/CAM technology that was originally developed to fabricate prototypes for industrial purposes, and the use of RP systems can reduce the sensitivity and the technical complexity involved in the creation of a dental prosthesis [35].

This in vitro study compared the internal and marginal precisions of different posterior FDPs manufactured using three different methods: a milling system, laser-assisted RP through selective laser melting (SLM), and the lost-wax casting technique. The aim was to quantify the differences in accuracy between copings produced with SLM and the other techniques so as to provide an experimental basis for clinical research. The hypothesis of the study is the objective of allowing the clinician to rely on technology in increasingly more reliable and reproducible way, with a minimum margin of error in the realization of FDPs.

## 2. Materials and Methods

In the study the following methods have been used: (1) the CAD/CAM Cercon system and the Compartis system (DeguDent, Hanau, Germany) for manufacturing Y-TZP (zirconium oxide stabilized with yttrium oxide) single copings and bridges using a milling technique, (2) SLM for Co-Cr alloy, and (3) the conventional lost-wax technique for the realization of single crowns and bridges in Ni-Cr alloy. The compositions of the materials selected for this study and their processing techniques are provided in Table 1.

### 2.1. Manufacturing the Models

**Figure 1:**
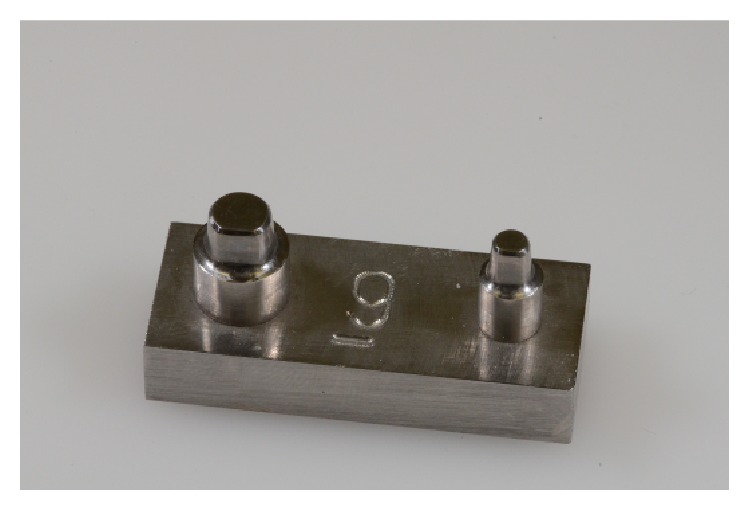
Master stainless-steel model.

A stainless-steel model with two abutments simulating a first premolar and a second molar screwed tightly on a holder (40 mm long, 16 mm wide, and 8 mm thick) was machined at the Mechanical and Aerospace Department of “Sapienza” University of Rome (Figure 1).

The abutments were positioned on the platform to receive posterior four-unit FDPs or two single crowns. The preparation had a finish line with a 1.0 mm radius, a 10-degree angle of convergence of the axial walls, and a shoulder margin, thereby simulating clinical conditions. A vertical flat surface parallel to the long axis of the abutments allowed the correct insertion of the structures and also ensured the stability of the platform. Specimens were measured using a digital caliper (Aura Dental, Germany) with an accuracy of 0.01 mm. The steel sample was used as the master model, and it was duplicated using silicone (Elite Double 22, Zhermack, Germany). The master steel model was placed in a rigid plastic container with a top opening, and the silicone material was then poured in, with the expected hardening times following the manufacturer's instructions. Casts of polyurethane resin were then made (K.W. New Color, Techim Group), which were used as working dies. Thirty resin casts were constructed, and they were divided into three groups: group 1, Ni-Cr alloy; group 2, Co-Cr alloy; and group 3, Y-TZP. Each group comprised five resin casts for single copings and five resin casts for four-unit bridges (*n* = 10/group). The resin casts with single copings were then dissected to analyze the copings separately. The same protocol was used for the bridges. All of the samples used in the study were marked with an identification code (*n* = 20), with 10 single structures and 10 elements of bridges being fabricated for each group.

### 2.2. Manufacturing the Framework

**Figure 2:**
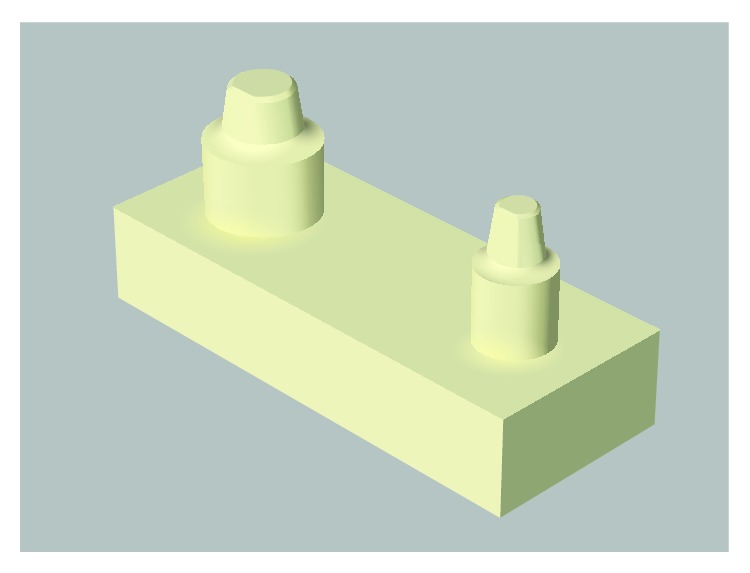
3D model.

The dies for copings in the Ni-Cr alloy group were coated with a layer of die spacer (total thickness of 20 *μ*m) applied 0.5 mm above the margin (Yeti Die Spacer, Yeti Dental, Engen, Germany). The Ni-Cr alloy was cast by a private dental laboratory (SaviDent, Rome, Italy) using the conventional lost-wax technique and single casting to fabricate the restorations. Wax patterns were prepared and invested with carbon-free phosphate bonded investment material (GC Stellavest, GC Corporation, Belgium) in accordance with the manufacturer's instructions (Figure 2). The patterns were casted in Ni-Cr alloy (Biologic NA, Conroe Dental, Ancona, Italy) using an induction and centrifugation casting machine (Seit Elettronica, Italy). After divesting, the castings were cleaned with 50 *μ*m Al_2_O_3_ using an air-borne-particle abrasion device (Basic Master, Renfert, Hilzingen, Germany). Each FDP was fabricated according to the manufacturer's instructions by the same dental technician. Y-TZP samples were digitized using the 3Shape D700 scanner (3S, DeguDent), which uses a laser-based optical scanning method. The 3S has a three-axis movement system that allows for an individualized scanning position of the casts. The CAD process was performed with the 3Shape Dental System software (3Shape A/S, Copenhagen, Denmark). All copings had a minimum thickness of 0.5 mm, which was consistent with the manufacturer's recommendation. All FDPs produced with the 3Shape system were fabricated in Y-TZP (Cercon Base, DeguDent) by a centralized milling center (Compartis, DeguDent) after transmitting the data via the Internet. Co-Cr alloy (StarLoy LS, DeguDent) copings were fabricated using the SLM technique. Scanning patterns and phase CAD were performed by the same method used for the Y-TZP samples. The production of substructures was outsourced to the Compartis Center (DeguDent).

All of the structures were repositioned on their models and then checked for the correct positioning. If the positioning was incomplete, the structure was adapted using a standardized protocol according to the literature and clinical practice [35–39]. Areas to be corrected were identified by applying a spray lacquer (Contact-Spray, Protechno, Girona, Spain). The colored spots inside the cap were removed with a tungsten carbide bur while using a water spray to clean the debris. The same dental expert adapted and verified all of the restorations.

### 2.3. Cementation Process

All FDPs were cemented using a conventional glass ionomer (Ketac Cem Easymix, 3M ESPE, USA), mixed following the manufacturer's instructions. The cement was placed on the axial surfaces of the abutments so that cementation would simulate the clinical procedure as closely as possible.

Each restoration was set on pillars and subjected to a pressure of 50 N [8, 40–43] for 10 minutes using a compression testing machine with automatic recognition of calibration data at 50 N (Mecmesin, United Kingdom).

### 2.4. Analyses and Measurements of Marginal Fit

**Figure 3:**
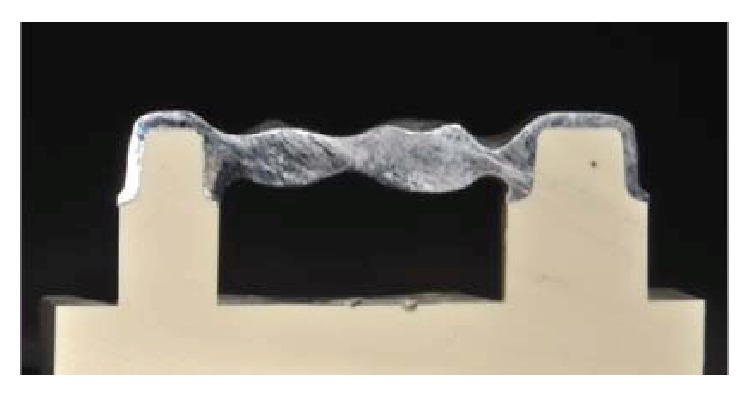
Sectioned framework.

**Figure 4:**
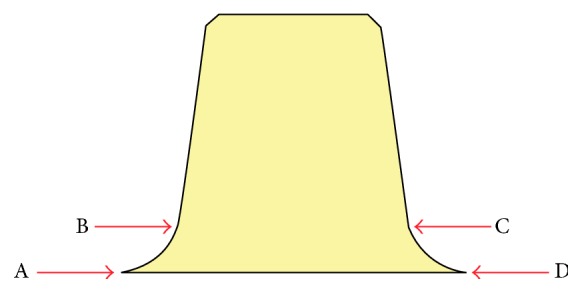
Representation of the four measuring points on each abutment.

At 24 hours after cementation, each framework was sectioned centrally in the mesiodistal direction (Figure 3) with the aid of a cutting machine (Micromet, Remet, Bologna, Italy). The fit of the substructures was evaluated as illustrated in Figure 4.

For each substructure, the four measurement locations were used to determine the precision of the marginal and internal fits: the marginal opening (points A and D), at the point of closest approximation between the model and the substructure, and the shoulder area (points B and C), corresponding to the internal adaptation of the substructure at 1 mm from the margin. Image analysis software (AxioVision Rel. 4.8, Zeiss, Germany) in combination with a stereomicroscope (Stemi 200-C, Zeiss) at ×60 magnification and a camera (AxioCam ICc1, Zeiss) were used for analyzing the marginal fit. The specimens were positioned in a base perpendicular to the optical axis of the microscope.

**Figure 5:**
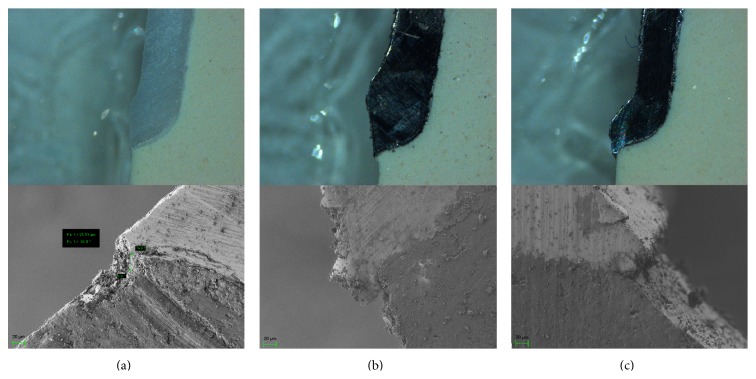
Stereomicroscope and SEM micrographs of the marginal precisions of (a) a Y-TZP premolar abutment, (b) a Co-Cr premolar abutment, and (c) an Ni-Cr alloy premolar abutment.

Given the thinness of the cement, additional micrographs were acquired using image analysis software (SmartSEM, Zeiss) in combination with field-emission scanning electron microscopy (SEM) at ×800 magnification (Auriga, Zeiss) at the Nanotechnology and Nanoscience Laboratory, SNN-Lab, Sapienza University of Rome, which was capable of performing low-voltage imaging of highly nonconductive materials. All measurements were performed by the same investigator, and the accelerating voltage was fixed at 1 kV (Figures 5(a), 5(b), and 5(c)) Measurements for each FDP were averaged, and these were used to determine the mean discrepancy in the marginal fit in each group (*n* = 20).

### 2.5. Statistical Analyses

The statistical analysis was carried out using Stata 12.0 software. Descriptive statistics included the calculation of mean ($\overset{-}{x}$) and standard deviation (SD) values for all available measurements at each point. A one-way analysis of variance (ANOVA) was carried out to identify statistically significant differences between the investigated systems in terms of marginal fit at the different measurement locations confirmed with the nonparametric Kruskal-Wallis test. A Bonferroni post hoc test was used to compare the different groups. The cutoff for significance was set at 5% in all tests.

## 3. Results

**Figure 6:**
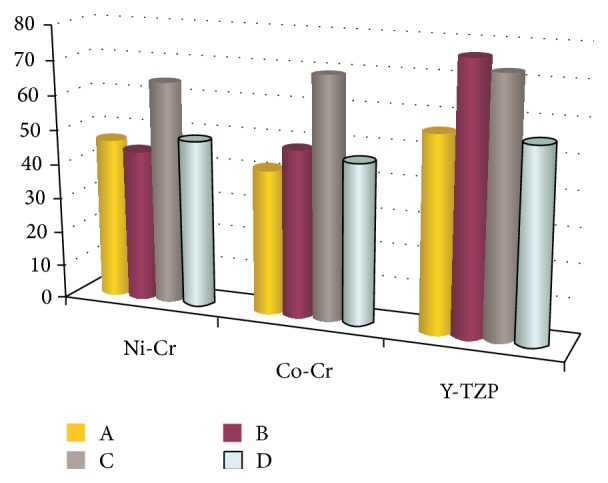
The total mean of marginal and internal discrepancies of 4 measurement points for each group (values in *μ*m).

The mean and SD values of the marginal and internal adaptations for all measurement points, tooth sizes, and production methods are presented in Tables 2 and 3. Measurements have been made in the same locations in stereomicroscope and SEM. The mean values for all measurement points are shown in Figure 6. The discrepancies were largest in the Y-TZP group. The discrepancies in tooth size between the premolar bridge, molar bridge, single premolar, and single molar were largest between the single premolar and the premolar bridge (*P* < 0.05).

Significant differences were present for the position parameter, with higher discrepancies at points A and B (*P* < 0.05). The best fit—independent of the different parameters—was at points A and D for the SLM technique and at points B and C for the lost-wax technique. The mean marginal discrepancy was 47.56 *μ*m for the Ni-Cr alloy, 55.6 *μ*m for Y-TZP, and 43.92 *μ*m for the Co-Cr alloy. The mean internal gap was 54.11 *μ*m for the Ni-Cr alloy, 74.73 *μ*m for Y-TZP, and 58.76 *μ*m for the Co-Cr alloy. ANOVA revealed statistically significant differences (*P* < 0.05) in marginal and internal adaptations among the groups at the four measurement points.

## 4. Discussion

RP technology has attracted enormous interest among researchers because it greatly facilitates the realization of bespoke three-dimensional (3D) objects. A focused high-power laser beam can selectively melt layers of metal alloy powder in a mass using thermal energy and so can be used to produce any desired 3D object under computer control. After each section is scanned, the thickness of the powder bed of an alloy of the base metal is lowered by one layer, and a new layer of metal-based alloy is applied on top. This process is repeated until the part is completed.

In addition, the remaining unprocessed powder can be reused, in contrast with conventional methods in which most of the material is wasted and there are spatial limitations restricting the production of complex shapes [44]. SLM technology is characterized by remarkable precision, the possibility of building virtually any required dental geometry, and a constant surface speed and therefore a high-quality milling (especially of undercuts) thanks to the availability of four-axis simultaneous milling.

The optimal clinical marginal gap remains controversial. McLean and von Fraunhofer [44] found that a prosthetic restoration is successful if the marginal gap is less than 120 *μ*m. Based on this criterion as the limit of clinical acceptance, the mean marginal and internal discrepancy values were clinically acceptable in all three groups in the present study.

While further research is necessary to optimize the process parameters and clinical applications, the laser-assisted RP procedure reported herein provides an efficient and rapid method for digitally designing and manufacturing complex metal structures for FDPs. It should be noted that many of the samples exhibited wide variations of the marginal gap; for example, while one surface was accurate to a few microns, there were large openings on the contralateral side. This may have been due to small displacements of the structure during corrections, which could have resulted in incomplete seating of the substructure and the largest marginal openings, or there may have been inaccuracies during cutting. All restorations were evaluated after the corrections had been made by the dental laboratory. Since the master model was duplicated in numerous resin models, there were many variables that could potentially change the results during the laboratory work, including the duplication time, small changes in water/powder ratio, water temperature, and wax distortion. In this study all of the substructures were placed and cemented in their respective resin models to test the differences between the different manufacturing techniques and the different materials used at the levels of the marginal gap and shoulder area.

The study was subject to several limitations.

1. All structures were adapted using a standardized protocol, and retouching was performed by the same technician in order to avoid large inaccuracies.
2. There was a possibility of samples being damaged during the cutting process. This risk was minimized but cutting under a water spray and using low feeding rates.
3. All samples were produced and tested under ideal conditions, which might not accurately reflect typical clinical use.
4. If the position of the framework was incomplete, the structure was adapted manually by technician using a standardized protocol with a margin of human error.

## 5. Conclusion

This study investigated the application of different techniques for the manufacture of FDPs. Within the limitations of this in vitro study, the following conclusions can be drawn.

1. Copings produced with SLM technology have better marginal adaptation within an acceptable range.
2. The type of metal alloy did not significantly affect the measurements.
3. The marginal and shoulder areas presented greater discrepancies in values between metal alloys and Y-TZP.
4. The cement gap was wider in the region of the shoulder than at the point of closure.
5. All of the techniques and materials tested resulted in acceptable marginal openings in vitro.

The RP technique is a substantial innovation for the manufacture of dental prostheses, allowing dentists to work more easily and faster while still ensuring the production of a high-quality finished product, due to significantly decreases in the risk of human error. It was concluded that, within the limitations of this study, the RP system can compete with conventional systems and can achieve a good marginal fit in vitro.

## Figures and Tables

**Table 1:** Composition and properties of materials used and their manufacturing processes.

Product	Process	Alloy composition (Wt %)	Elastic modulus (GPa)	Vickers hardness (Kg/mm²)	Yield strength (MPa, 0.2%)	Thermal expansion coefficient 10^−6^ *µ*m/mK (25–500°C)
Cercon base (DeguDent) Ni-Cr alloy	Lost wax casting	Co 0.1 Cr 14–16 Al 1–3 Ni 71–75 Mo 8–10 Be 0.1–1.9 Traces of Ti	218	360	586	13.9

StarLoy LS (DeguDent) Co-Cr alloy	Selective laser melting (SLM)	Co 55.2 Cr 18.4 W 18.4 Fe 6.0 Al 2.0	210	390	938–1024	14.3

Biologic NA (Conero Dental) Y-TZP	Milling	ZrO_2_ Y_2_O_3_ 5% HfO_2_ <2% Al_2_O_3_ and SiO_2_ <1%	210	1200	900–1200	10.5

**Table 2:** Mean values ($\overset{-}{x}$) and standard deviation values (SD) of points A and B for all abutments in each group (values in *μ*m).

Point A	Y-TZP $\overset{-}{x}$ (±SD)	Co-Cr $\overset{-}{x}$ (±SD)	Ni-Cr $\overset{-}{x}$ (±SD)	pV
Premolar abutment	51.37 (10.62)	43.88 (15.25)	48.49 (15.12)	0.696
Molar abutment	60.88 (6.56)	47.06 (18.96)	58.92 (15.20)	0.302
Single abutment	56.37 (9.09)	33.08 (7.56)	31.53 (5.30)	0.001
Single abutment	56.03 (5.45)	43.80 (17.26)	43.68 (21.68)	0.571

Point B	Y-TZP $\overset{-}{x}$ (±SD)	Co-Cr $\overset{-}{x}$ (±SD)	Ni-Cr $\overset{-}{x}$ (±SD)	pV

Premolar abutment	78.62 (18.25)	39.61 (12.90)	27.23 (16.18)	0.001
Molar abutment	75.47 (20.00)	73.60 (24.54)	69.64 (20.19)	0.911
Single abutment	77.87 (12.86)	30.79 (9.60)	36.08 (21.95)	0.002
Single abutment	74.25 (23.33)	48.65 (19.06)	42.07 (21.52)	0.181

**Table 3:** Mean values ($\overset{-}{x}$) and standard deviation values (SD) of points C and D for all abutments in each group (values in *μ*m).

Point C	Y-TZP $\overset{-}{x}$ (±SD)	Co-Cr $\overset{-}{x}$ (±SD)	Ni-Cr $\overset{-}{x}$ (±SD)	pV
Premolar abutment	52.02 (24.20)	77.60 (32.55)	73.87 (21.49)	0.404
Molar abutment	96.98 (17.13)	93.26 (43.39)	79.03 (18.05)	0.597
Single abutment	57.72 (11.21)	44.12 (15.96)	40.40 (16.38)	0.262
Single abutment	79.24 (17.01)	63.15 (22.38)	60.69 (26.21)	0.488

Point D	Y-TZP $\overset{-}{x}$ (±SD)	Co-Cr $\overset{-}{x}$ (±SD)	Ni-Cr $\overset{-}{x}$ (±SD)	pV

Premolar abutment	45.94 (8.05)	46.42 (17.61)	40.75 (10.56)	0.743
Molar abutment	60.02 (9.48)	53.66 (18.87)	64.11 (18.28)	0.600
Single abutment	56.74 (29.73)	36.51 (13.09)	50.27 (6.92)	0.292
Single abutment	58.48 (22.72)	48.18 (26.27)	35.70 (21.53)	0.427
